# Composition and characteristics of soil microbial communities in cotton fields with different incidences of Verticillium wilt

**DOI:** 10.1080/15592324.2022.2034271

**Published:** 2022-02-17

**Authors:** Yun Zhang, Yuanxue Yang, Lang Yu, Aiyu Wang, Chao Xue, Jianhua Zhang, Ailing Duan, Ming Zhao

**Affiliations:** a Institute of Industrial Crops, Shandong Academy of Agricultural Sciences, Jinan, China; b Plant Protection Station of Xinjiang Uygur Autonomous Region, Urumqi, China

**Keywords:** microbial community, Verticillium wilt, fungi, bacteria

## Abstract

Soil microorganisms could affect the growth of plants and play an important role in indicating the change of soil environment. Cotton Verticillium wilt is a serious soil borne disease. This study aimed to analyze the community characteristics of soil microorganisms in cotton fields with different incidences of Verticillium wilt, so as to provide theoretical guidance for the prevention and control of soil borne diseases of cotton. Through the analysis of soil microbial communities in six fields, the results showed that there was no difference in fungal and bacterial alpha-diversity index before cotton planting, while there were differences in rhizosphere of diseased plants. For fungal beta diversity indexes, there were significant differences in these six fields. There was no significant difference for bacterial beta diversity indexes before cotton planting, while there was a certain difference in the rhizosphere of diseased cotton plants. The composition of fungi and bacteria in different fields was roughly the same at the genus level, but the abundances of the same genus varied greatly between different fields. Before cotton planting, there were 61 fungi (genera) and 126 bacteria (genera) with different abundances in the six fields. *Pseudomonas, Sphingomonas* and *Burkholderia* had higher abundances in the fields with less incidence. This study will provide a theoretical basis for microbial control of Cotton Verticillium wilt.

## 1. Introduction

Cotton Verticillium wilt, caused by the soil-borne fungus *Verticillium dahliae* Kleb., detrimentally affects cotton yield and fiber quality, and is a very serious plant disease on cotton all over the world.^1^ The pathogen can survive for a long time in the form of microsclerotia in soil., and it can infect a variety of plants like eggplant, potato, lettuce, olive, and sunflower.^2–6^ Therefore, it is difficult to prevent and control.^7^ At present, there is no ideal control method.

Plant microbiome is composed of a variety of microorganisms, which is called the second genome of plants. Plant root microorganisms are diverse, with a number of about tens of thousands of species.^8^ It is increasingly agreed that plants and soil microorganisms are closely linked through resources, physical microhabitats, nutrient dynamics and other environmental conditions.^9^ There are many beneficial microorganisms in plant rhizosphere soil. Beneficial soil microbes contribute to promoting plant growth,^10^ and pathogen resistance.^8,11^ In return, plants secrete fixed carbon and nitrogen to the rhizosphere, thereby supporting the microbial community.^12^ Some studies have shown that regulating the ecological balance of soil microorganisms is helpful to inhibit crop diseases.

The microbial community structure is one of the indicators of soil condition. Interactions between plants and soil microbes are important for plant growth and resistance.^13^ Many studies have shown that soil microbial structure could affect the occurrence of plant diseases. In susceptible and resistant cotton varieties, the relative abundance of many rhizosphere and endophytic microorganisms was different, and the relative abundance of beneficial microbes were higher in resistant cultivars, such as *Bacillales, Pseudomonadales, Rhizobiales*, and *Trichoderma*;^14^ the bacterial alpha-diversity in rhizosphere of diseased cotton plants was lower than that in healthy cotton plants; many rhizospheric microorganism differed in their relative abundance between diseased and healthy cotton plants;^7^ the number and abundance of operational taxonomic units (OTUs) in the soil of naturally seriously diseased cotton fields were higher than those in lightly diseased or disease-free fields, while the diversity of fungi was low in Xinjiang.^15^ The effects of plants on soil microbiome varied greatly, and plant growth and resistance to pathogens were related to complex soil microbial communities.^13^ At present, several studies have investigated on soil fungal and bacterial microbial communities with different incidences of Cotton Verticillium wilt.^15^ The difference of soil microorganisms before planting is not clear. Therefore, the purpose of this study was mainly to report the differences of soil microorganisms before planting and rhizosphere microorganisms of diseased plants in fields with different disease degrees.

## 2. Materials and methods

### 2.1 Cite description

Six monoculture cotton fields (100 m apart) with the incidence of Verticillium wilt were used for experiment in 2018 in Xiajin(XJ), Linqing(LQ), Juye(JY), Lijin(LJ), Jinxiang(JX), Huimin(HM), Shandong Province, China. The cotton cultivar was Lumian 1141.

### 2.2 Investigation on cotton Verticillium wilt

At the peak of Cotton Verticillium wilt, August 26, 2018, Cotton Verticillium wilt was investigated in 6 monoculture cotton fields, HM, LJ, JY, JX, XJ and LQ, respectively. The diagonal 5-point survey method was adopted, with an interval of 10 m between the two points, and 50 plants were continuously investigated at each point. The disease index was investigated according to the 5-level classification standard, and the classification standard was referred to the method Wei et al.,^14^ level 0: no symptoms, level 1: incidence area ≤ 33%, level 2: 33% < incidence area ≤ 66%, level 3: 66% < incidence area ≤ 99%, level 4: incidence area is 100%, and the disease index (DI) of each plot was calculated, DI = (0·n_0_ + 1·n_1_ + 2·n_2_ + 3·n_3_ + 4·n_4_)/(4·n) × 100%. n_0_-n_4_ is the number of plants with corresponding disease grade (0–4), and n is the total number of plants investigated.

### 2.3 Soil samples collection

About April 20, 2018, before cotton planting, soil samples 10–20 cm away from the soil surface were taken from the six fields where Cotton Verticillium wilt was investigated above. Three samples were taken from each filed at an interval of 30 m. Each sample was taken five times in 1 m^2^ to mix the soil. A total of 18 samples were taken as the control (HM1, LJ1, JY1, JX1, XJ1 and LQ1). In the period of Cotton Verticillium wilt, the rhizosphere soil of diseased cotton plants was taken. 2 mm rhizosphere soil attached to the root system of cotton plants with Verticillium wilt was collected by shaking off method.^7^ Three samples were taken from each field with an interval of 30 m. Five plants were taken from each sampling point, and roots were first shaken to remove loosely adhering soil particles, then the roots were cut into pieces of 2-cm length. Rhizosphere samples were collected in aliquots of 30-g roots in 1:50 TE buffer by shaking, filtering, and centrifuging. A total of 18 samples were taken (HM4, LJ4, JY4, JX4, XJ4 and LQ4).

### 2.4 DNA extraction and qPCR

The soil samples were collected to extract DNA according to the FastDNA^TM^ SPIN Kit (MP Biomedicals, USA)for Soil extraction specification, and agarose gel electrophoresis was used to detect DNA quality. DNA concentration detector One drop 2000 (Thermo, USA) was used to determine the concentration of the DNA. The bacterial 16S rRNA sequencing fragment was V3-V4, and the regional universal primer was 338 F:5’-ACTCCTACGGGAGGCAGCAG-3’, 806 R: 5’-GGACTACHVGGGTWTCTAAT-3’,^16^ the fungal ITS sequencing fragment was ITS2, and the regional universal primer was fITS7: 5’-GTGARTCATCGAATCTTTG-3’, ITS4: 5’-TCCTCCGCTTATTGATATGC-3’,^17^ PCR reaction system and program were performed formed following a previous study.^18^ PCR products were purifed with Gel Extraction Kit (Vazyme, China) and pooled in equimolar concentrations. Paired-end sequencing (PE300) of bacterial and fungal amplicons were carried out on an Illumina MiSeq platform at Hangzhou Lianchuan Biotechnology Co., Ltd (Hangzhou, China).

### 2.5 Statistical analysis

Fqtrim and Vsearch (v2.3.4) were used to filter the low-quality sequences and the chimera. After noise reduction with DADA2, the feature table and feature sequence are obtained. According to 97% sequence similarity, the sequences obtained above were clustered into OTUs. After OTU clustering, the OTU abundance (number of sequences) in each sample was counted, and the OTU whose abundance value was lower than 0.001% of the total sequencing amount of all samples was removed to ensure the accuracy of the analysis data. Classify the OTU sequences in the fungal ITS database SILVA and unite, and then difference analysis were conducted based on the OTU abundance (*p* ≤ .05). For species annotation, the feature-classifier was used for sequence alignment. Alpha-diversity and beta diversity were normalized by flattening, and species annotation was normalized by relative abundance. Alpha-diversity and beta diversity were analyzed with QIIME 2.^19^ Alpha-diversity analyses included observed species (Sobs), Shannon, Simpson and Chao1. Beta diversity was calculated by weighted UniFrac distance and analyzed by Principal coordinate analyses (PCoA).^20^ The diferences of disease index and alpha-diversity index were determined by one-way analysis of variance (ANOVA), and Tukey’s HSD method was used for test.

## 3. Results

### 3.1 Occurrence degree of Verticillium wilt in different fields

**Figure 1.**
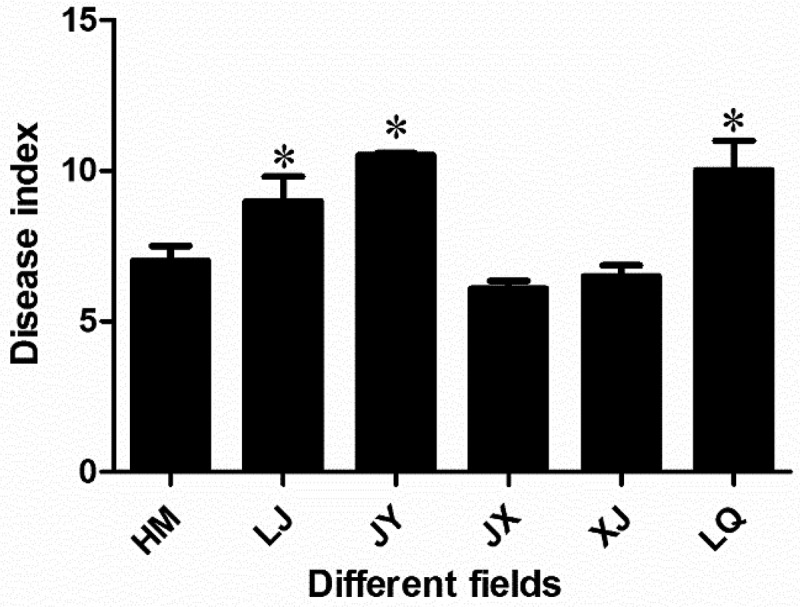
Disease index of Cotton Verticillium wilt in different fields. * indicated that there was significant difference between different groups at the level of *P* < .05. The bar chart showed the mean ± standard deviation.

In 2018, the disease indexes of HM, LJ, JY, JX, XJ and LQ were 7.00, 8.99, 10.50, 6.08, 6.50 and 10.01, respectively. The disease indexes of LJ, JY and LQ were significantly higher than that of the other three fields, and the incidence of Verticillium wilt was more serious. The results showed that the incidence of Verticillium wilt in JY, LJ and LQ was relatively heavy, while that in JX, XJ and HM was relatively light (Figure 1).

### 3.2 Operational taxonomic units (OTUs)

**Figure 2.**
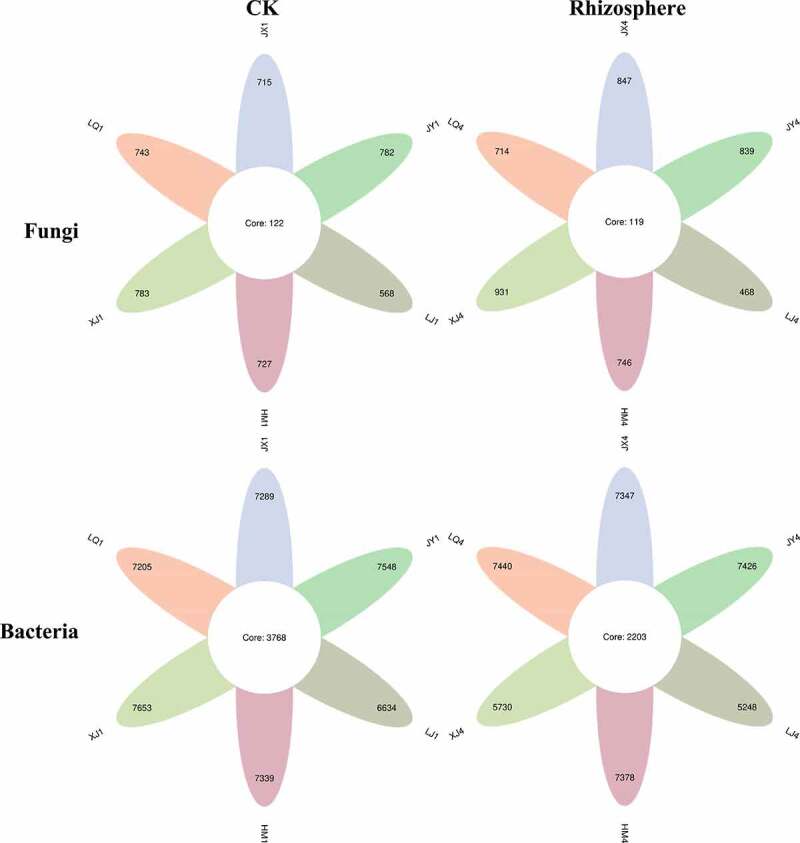
The number of OTUs in six fields(*P* < .05). CK represents the soil sample before cotton planting, and Rhizosphere represent the rhizosphere soil samples of diseased plants with Verticillium wilt.

Before cotton planting, there were 122 common fungal OTUs in the soil of the six fields, and XJ have the largest number of OTUs (783), LJ (568) was the least; the common bacterial OTUs in the six fields were 3768, the number of OTUs in XJ (7653) was the largest, and the least was in LJ (6634). In the period of Cotton Verticillium wilt, the common fungal OTUs in the rhizosphere of diseased plants in six fields were 119, the number of OTUs in XJ (931) was the largest, and the least was in LJ (468); the common bacterial OTUs in the rhizosphere of diseased plants in six fields was 2203, the number of OTUs in LQ (7440) was the largest, and the least was in LJ (5248) (Figure 2).

### 3.3 Alpha diversity

Before cotton planting in these six fields, the fungal alpha-diversity indexes Sobs, Shannon and Simpson in LQ and JY fields were relatively high, but there was no significant difference among the six fields. The alpha-diversity index Sobs of rhizosphere fungi of diseased plants in XJ and JX fields were significantly higher than that in LJ field, and there was no significant difference in Shannon and Simpson indexes (Table 1). Before cotton planting, the bacterial alpha-diversity indexes Sobs and Shannon of XJ and HM fields were relatively high, but the difference was not obvious; the bacterial alpha-diversity indexes Sobs and Shannon of rhizosphere of diseased plants in HM field were significantly higher than that in LJ field (Table 2).

**Table 1.** The fungal diversity in different fields and different periods.

Fields	CK			Rhizosphere		
	Sobs	Shannon	Simpson	Sobs	Shannon	Simpson
JX	336.00 ± 32.08a	5.00 ± 0.47a	0.91 ± 0.06a	420.67 ± 17.04ab	5.68 ± 0.59a	0.95 ± 0.02a
LQ	351.00 ± 39.96a	5.41 ± 0.23a	0.94 ± 0.02a	313.00 ± 50.09bc	4.24 ± 0.60a	0.85 ± 0.07a
XJ	346.33 ± 13.32a	4.85 ± 1.07a	0.87 ± 0.14a	487.00 ± 52.09a	5.95 ± 0.30a	0.96 ± 0.01a
HM	343.00 ± 5.00a	4.99 ± 0.12a	0.92 ± 0.01a	351.33 ± 81.64abc	5.11 ± 0.80a	0.92 ± 0.05a
LJ	272.67 ± 68.71a	4.64 ± 0.74a	0.87 ± 0.09a	221.33 ± 41.48c	4.24 ± 0.77a	0.84 ± 0.08a
JY	355.67 ± 66.25a	5.33 ± 0.40a	0.92 ± 0.03a	402.67 ± 53.15ab	5.51 ± 0.59a	0.94 ± 0.03a

Data are mean ± SE. Different letters indicate significant difference at the level of *p* < 0.05.

**Table 2.** The bacterial diversity in different fields and different periods.

Fields	CK			Rhizosphere		
	Sobs	Shannon	Simpson	Sobs	Shannon	Simpson
JX	3622.33 ± 270.78a	10.45 ± 0.18a	1.00 ± 0.00a	3827.67 ± 246.78ab	10.57 ± 0.23ab	1.00 ± 0.00a
LQ	3707.33 ± 277.39a	10.62 ± 0.15a	1.00 ± 0.00a	3929.67 ± 223.34ab	10.57 ± 0.19ab	1.00 ± 0.00a
XJ	3991.00 ± 103.79a	10.64 ± 0.14a	1.00 ± 0.00a	3564.67 ± 343.27bc	10.51 ± 0.31ab	1.00 ± 0.00a
HM	3808.67 ± 65.31a	10.49 ± 0.09a	1.00 ± 0.00a	4302.00 ± 206.65a	10.88 ± 0.13a	1.00 ± 0.00a
LJ	3109.00 ± 863.41a	9.98 ± 0.76a	1.00 ± 0.00a	3023.67 ± 283.68c	9.96 ± 0.39b	1.00 ± 0.00a
JY	3655.00 ± 321.31a	10.27 ± 0.47a	1.00 ± 0.00a	3961.00 ± 103.50ab	10.48 ± 0.16ab	1.00 ± 0.00a

Data are mean ± SE. Different letters indicate significant difference at the level of *p* < 0.05.

### 3.4 Beta diversity index

**Figure 3.**
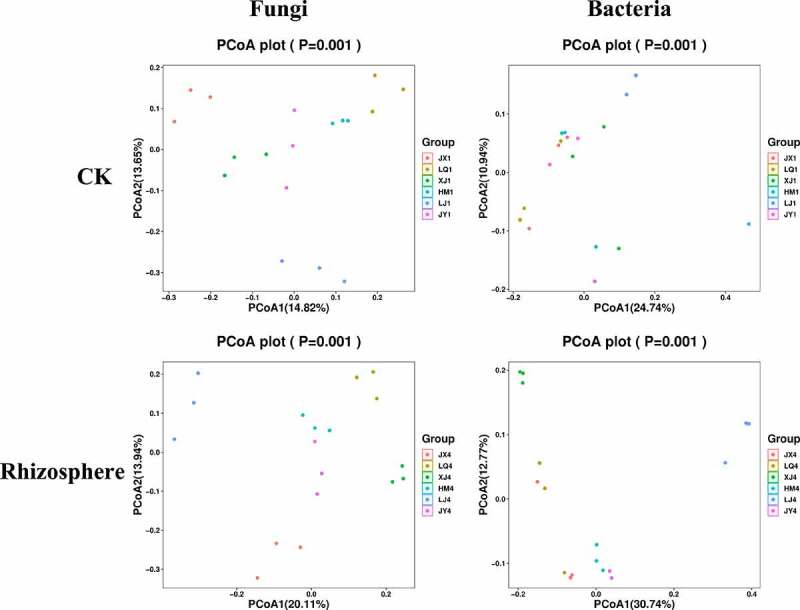
PCoA analysis of bacterial and fungal communities in the six fields. ITS and 16S amplicon sequences were used to profile bacterial and fungal communities, respectively. CK represents the soil sample before cotton planting, and Rhizosphere represent the rhizosphere soil samples of diseased plants with Verticillium wilt.

In contrast to the alpha-diversity indexes, PCoA analysis based on OTU level showed that beta diversity indexes of fungi showed differences in these six different fields, while the difference in beta diversity indexes of bacteria was not very obvious; Adonis analysis showed that there were significant differences among different samples in the same field. The beta diversity index also showed some differences in rhizosphere bacteria of diseased plants in different fields (Figure 3).

### 3.5 Taxonomy information

**Figure 4.**
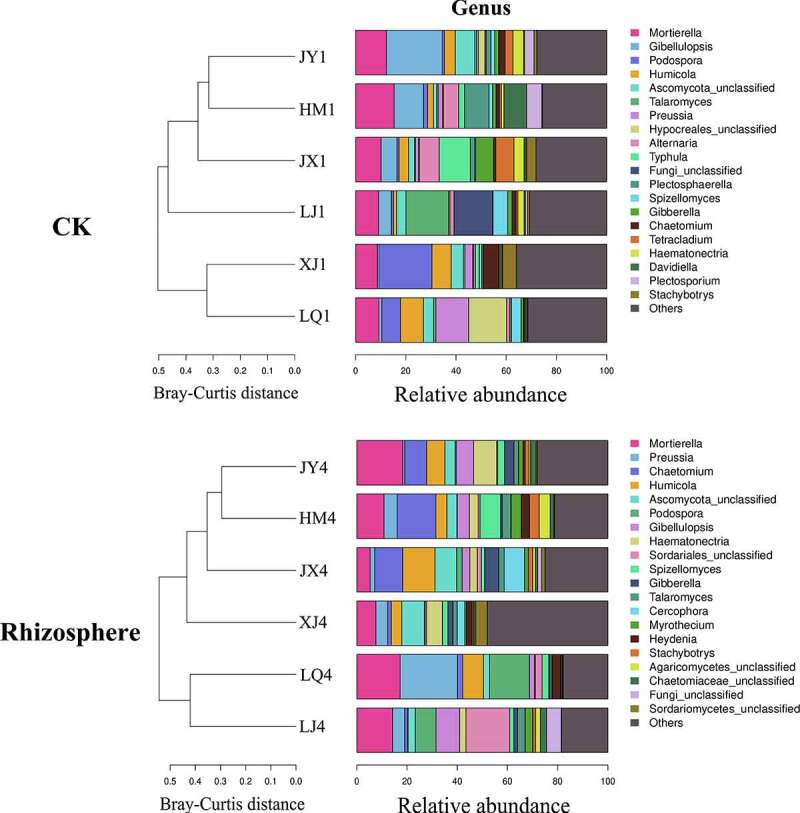
Composition of fungi in soil of different fields at genus level. CK represents the soil sample before cotton planting, and Rhizosphere represent the rhizosphere soil samples of diseased plants with Verticillium wilt.

The composition of fungal microorganisms in different fields was roughly the same at the genus level, but the abundance of the same genus varied greatly between different fields. The composition of fungi in the rhizosphere of diseased plants have changed compared with pre planting, of which 7 genera have changed from *Alternaria, Hypocreales, Typhula, Plectosphaerella, Tetraclaudium, Davidella* and *Plectosporium* to *Sordariales, Cercophora, Myrothecium, Heydenia, Agaricomycetes, Chaetomiaceae* and *Sordariomycetes*, the other 13 genera remained unchanged (Figure 4). Before planting, *Chaetomium*, a beneficial microorganism, had the highest abundance in XJ field, reaching 6.23. The abundances of HM, JX, JY, LJ and LQ were 1.29, 0.90, 2.55, 1.68 and 0.22, respectively (Table 3). *Chaetomium* in rhizosphere of Cotton Verticillium wilt diseased plants had the highest abundance in HM field, reaching 15.48. The abundances of JX, JY, LJ, LQ and XJ were 11.18, 8.68, 1.16, 2.13 and 1.40, respectively (Table 4).

**Table 3.** Composition of fungi in soil before cotton planting at the genus level.

Genus	HM1	JX1	JY1	LJ1	LQ1	XJ1
*Mortierella*	15.35	10.17	12.27	9.11	9.29	8.69
*Gibellulopsis*	11.71	6.32	22.22	5.12	1.18	0.61
*Podospora*	1.67	0.79	0.91	0.88	7.44	21.14
*Humicola*	2.30	3.83	4.28	1.20	9.14	7.61
*Ascomycota*	1.31	2.37	7.82	3.76	4.03	4.90
*Talaromyces*	0.53	0.33	0.72	17.04	0.89	0.63
*Preussia*	1.80	1.23	0.68	0.04	13.11	3.12
*Hypocreales*	0.40	0.41	2.66	0.43	15.10	0.32
*Alternaria*	5.96	7.82	0.40	1.57	1.27	0.70
*Typhula*	2.44	12.45	0.00	0.00	0.00	1.51
*Fungi*	0.00	0.03	0.05	15.42	0.00	0.00
*Plectosphaerella*	9.68	1.71	1.91	0.15	0.66	0.02
*Spizellomyces*	1.45	0.41	1.51	5.87	3.78	0.93
*Gibberella*	1.38	7.11	1.57	1.71	0.91	0.57
*Chaetomium*	1.29	0.90	2.55	1.68	0.22	6.23
*Tetracladium*	0.76	7.23	3.16	0.68	0.30	0.37
*Haematonectria*	1.04	3.97	4.15	2.44	0.36	0.45
*Davidiella*	9.13	0.74	0.43	0.50	0.40	0.57
*Plectosporium*	5.96	0.42	3.77	0.90	0.37	0.20
*Stachybotrys*	0.30	3.64	1.16	0.76	0.23	5.43
Others	25.55	28.13	27.78	30.72	31.31	36.00

**Table 4.** Composition of fungi in rhizosphere of diseased plants at the genus level.

Genus	HM4	JX4	JY4	LJ4	LQ4	XJ4
*Mortierella*	10.85	5.30	18.36	14.24	17.27	7.57
*Preussia*	5.17	1.82	0.77	4.85	22.80	4.73
*Chaetomium*	15.48	11.18	8.68	1.16	2.13	1.40
*Humicola*	4.41	12.80	7.32	0.21	8.24	4.24
*Ascomycota*	3.93	8.66	4.04	2.79	2.42	8.98
*Podospora*	0.15	2.18	0.45	8.28	15.94	0.31
*Gibellulopsis*	4.87	3.10	6.93	9.43	1.95	0.58
*Haematonectria*	3.48	2.93	9.18	2.54	0.36	6.20
*Sordariales*	0.71	1.58	0.37	17.43	2.70	0.17
*Spizellomyces*	8.20	1.39	2.76	1.60	2.71	2.08
*Gibberella*	0.66	5.59	3.77	1.49	0.49	2.03
*Talaromyces*	3.45	2.17	1.77	2.97	0.69	1.67
*Cercophora*	0.12	8.09	0.05	0.06	0.02	3.10
*Myrothecium*	3.99	1.58	1.77	3.02	0.14	0.60
*Heydenia*	3.34	0.00	0.82	0.06	3.24	2.24
*Stachybotrys*	3.82	1.70	1.60	1.10	0.15	0.43
*Agaricomycetes*	4.39	1.09	0.58	1.86	0.34	0.33
*Chaetomiaceae*	1.46	0.90	2.25	2.47	0.40	0.72
*Fungi_unclassified*	0.00	1.59	0.00	5.86	0.00	0.00
*Sordariomycetes*	0.29	1.30	0.47	0.11	0.21	4.60
Others	21.22	25.06	28.09	18.46	17.79	48.01

**Figure 5.**
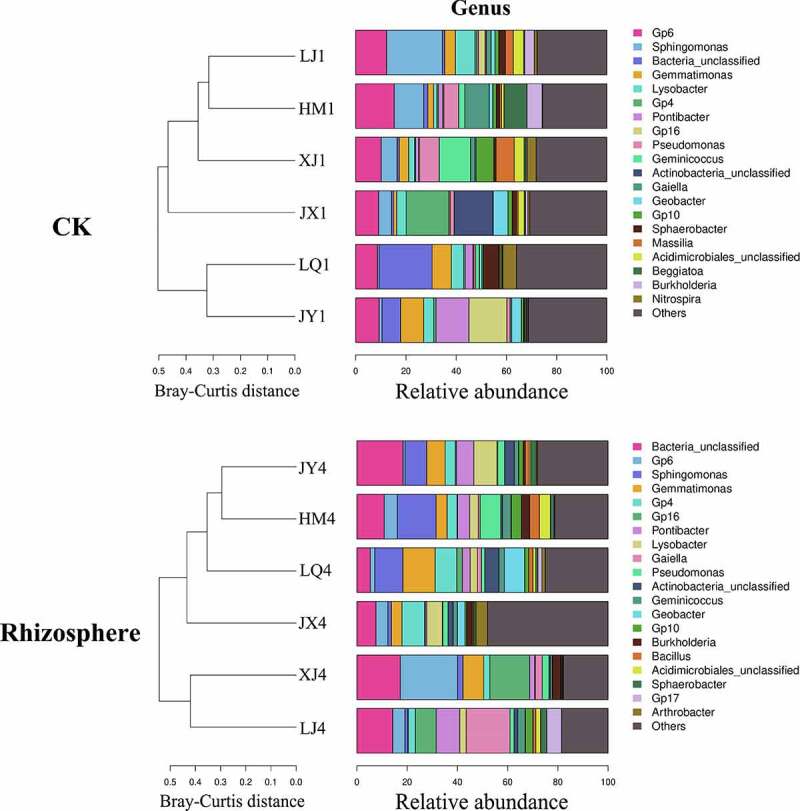
Composition of bacteria in soil of different fields at genus level. CK represents the soil sample before cotton planting, and Rhizosphere represent the rhizosphere soil samples of diseased plants with Verticillium wilt.

The composition of bacterial microorganisms in different fields was also roughly the same at the genus level, but the abundance of the same genus varied greatly between different fields (Figure 5). The composition of bacteria in the rhizosphere of diseased plants have changed compared with pre planting, of which 3 genera have changed from *Massilia, Beggiatoa* and *Nitrospira* to *Bacillus, Gp17* and *Arthrobacter*, and the other 17 genera remained unchanged. Before planting, the total abundances of beneficial microorganisms *Pseudomonas* and *Burkholderia* were the highest in LQ field, reaching 4.49, and the total abundances of HM, JX, JY, LJ and XJ were 1.74, 3.69, 3.46, 1.55 and 4.07, respectively (Table 5). *Pseudomonas, Bacillus* and *Burkholderia* were beneficial microorganisms in the rhizosphere of diseased plants, and the total abundances of them were the highest in LQ field, reaching 3.87. The total abundances of HM, JX, JY, LJ and XJ were 2.85, 3.72, 3.39, 3.77 and 3.35, respectively (Table 6).

**Table 5.** Composition of bacteria in soil before cotton planting at the genus level.

Genus	HM1	JX1	JY1	LJ1	LQ1	XJ1
*Gp6*	5.70	9.01	7.21	4.64	10.58	8.01
*Sphingomonas*	9.16	7.24	5.50	8.69	4.93	4.60
*Bacteria*	4.47	6.22	7.05	6.85	6.70	7.80
*Gemmatimonas*	5.04	5.33	5.93	4.99	6.86	5.72
*Lysobacter*	5.10	4.73	4.36	2.22	2.96	4.18
*Gp4*	2.07	4.23	2.63	1.83	5.21	3.97
*Pontibacter*	1.34	2.14	4.79	6.38	0.72	1.57
*Gp16*	2.30	3.16	1.94	1.50	2.99	2.68
*Pseudomonas*	1.10	2.87	2.62	1.27	3.19	3.32
*Geminicoccus*	1.25	1.61	2.29	2.10	1.70	2.91
*Actinobacteria*	1.25	1.33	1.89	2.01	1.57	1.83
*Gaiella*	1.40	1.81	1.46	1.00	1.90	1.73
*Geobacter*	1.04	1.24	1.93	1.17	1.69	1.77
*Gp10*	0.79	1.26	1.20	1.26	1.40	1.56
*Sphaerobacter*	1.01	1.12	1.38	1.61	0.98	1.27
*Massilia*	1.39	1.36	1.77	1.42	0.47	0.24
*Acidimicrobiales*	0.89	0.84	0.86	0.74	0.89	0.76
*Beggiatoa*	0.36	0.83	1.21	0.38	1.07	0.90
*Burkholderia*	0.64	0.82	0.84	0.28	1.30	0.75
*Nitrospira*	0.55	0.71	0.76	0.54	1.04	1.00
Others	53.18	42.14	42.39	49.11	41.86	43.43

**Table 6.** Composition of bacteria in rhizosphere of diseased plants at the genus level.

Genus	HM4	JX4	JY4	LJ4	LQ4	XJ4
*Bacteria*	9.21	6.56	8.93	9.77	6.67	6.12
*Gp6*	7.29	9.91	8.42	3.55	9.14	8.88
*Sphingomonas*	6.48	5.13	6.23	7.74	7.15	3.76
*Gemmatimonas*	6.59	6.81	5.26	3.28	5.94	6.15
*Gp4*	3.49	6.29	4.99	1.55	5.28	8.56
*Gp16*	3.03	3.42	3.49	1.67	2.99	4.32
*Pontibacter*	1.73	0.84	4.30	7.20	1.61	0.23
*Lysobacter*	2.51	2.80	2.81	1.60	3.78	1.09
*Gaiella*	1.57	2.23	1.99	0.88	1.97	3.15
*Pseudomonas*	1.38	1.62	1.62	0.88	1.69	1.49
*Actinobacteria*	1.54	1.50	1.39	1.23	1.22	1.60
*Geminicoccus*	1.37	1.59	1.52	1.50	1.28	0.67
*Geobacter*	1.55	1.29	1.28	1.15	1.23	1.32
*Gp10*	0.76	1.36	1.00	1.54	1.39	1.12
*Burkholderia*	1.00	1.54	0.92	0.15	1.44	1.35
*Bacillus*	0.47	0.56	0.85	2.74	0.74	0.51
*Acidimicrobiales*	0.98	1.04	0.79	0.69	0.82	1.41
*Sphaerobacter*	0.91	0.63	0.81	1.07	0.62	1.16
*Gp17*	0.58	0.91	0.56	0.11	1.09	1.73
*Arthrobacter*	0.86	0.64	0.60	0.30	1.03	1.39
Others	46.70	43.29	42.24	51.38	42.92	43.98

### 3.6 Fungi and bacteria with significant differences in abundances

Before cotton planting, there were 61 fungi with significant differences in abundances in the six fields (*p* < .05), including *Gibellulopsis, Podospora, Preussia, Plectosporium, Haematonectria, Plectosphaerella, Stachybotrys, Acremonium, Wardomyces, Heydenia, Fusarium*, etc. *Fusarium* fungi are pathogens of many diseases. During the onset of Cotton Verticillium wilt, there were 65 fungi with significant differences in abundances in rhizosphere(*p* < .05), including *Podospora, Haematonectria, Cercophora, Pseudonymnoascus, Fusarium, Metacordyceps, Tetraclaudium, Acromonium, Dokmaia, Emericellopsis*, etc (Table 7).

**Table 7.** Fungi and bacteria with significant differences in abundances in the six fields at the genus level (Top 15).

Fungi(genus)		Bacteria(genus) JX4	
CK Rhisphere	Rhisphere	CK	Rhisphere
*Dokmaia*	*Apiosordaria*	*Salinimicrobium*	*Iamia*
*Cephalotrichum*	*Acremonium*	*Albidovulum*	*Pelagibius*
*Plectosporium*	*Aleuria*	*Rhodothermaceae*	*Desulfoglaeba*
*Heydenia*	*Fusarium*	*Dehalococcoides*	*Arthrobacter*
*Stachybotrys*	*Cephalotrichum*	*Geminicoccus*	*Pontibacter*
*Acremonium*	*Heydenia*	*Aciditerrimonas*	*Conexibacter*
*Haematonectria*	*Metacordyceps*	*Sphingomonas*	*Burkholderia*
*Wardomyces*	*Tetracladium*	*Beggiatoa*	*Sphaerobacter*
*Gibellulopsis*	*Haematonectria*	*Caldilinea*	*Gaiella*
*Preussia*	*Cercophora*	*Xanthomonadaceae*	*Solirubrobacter*
*Hypocreales*	*Cladorrhinum*	*Deltaproteobacteria*	*Gp10*
*Typhula*	*Schizothecium*	*Pseudomonas*	*Gp17*
*Plectosphaerella*	Hypocreales	Desulfoglaeba	Anaerolineaceae
*Microascaceae*	Dokmaia	Arthrobacter	Bacteria
*Podospora*	Pseudogymnoascus	Nannocystis	Paenisporosarcina

CK represents the soil samples before cotton planting, and Rhizosphere represent the rhizosphere soil samples of diseased plants. The difference was analysis at *p* < 0.05.

Before cotton planting, there were 126 bacteria with significant differences in abundances in the six fields(*p* < .05), mainly including *Sphingomonas, Pseudomonas, Rhizorhapis, Geminicoccus, Beggiatoa, Caldilinea, Desulfoglaeba, Arthrobacter, Albidovulum, Aciditerrimonas, Dehalococcoides*, etc (Table 7). Among them, the abundance of *Pseudomonas* from high to low was XJ (3.32), LQ (3.19), JX (2.87), JY (2.62), LJ (1.27) and HM (1.10) (Table 8); the abundance of the highest field was 3.02 times that of the lowest field, and the abundance of XJ and JX fields were higher with mild disease. The abundances of *Sphingomonas* in HM and JX fields were 9.16 and 7.24, and they were significantly higher than that of LQ and JY fields with heavy disease. The abundances of *Rhizorhapis* were LJ (0.03), XJ (0.02), JX (0.01), LQ (0.01), HM (0.01), and JY (0.00) from high to low (Table 8). During the onset of Cotton Verticillium wilt, there were 195 bacteria with significant differences in abundances in rhizosphere(*p* < .05), such as *Pontibacter, Gaiella, Gp10, Burkholderia, Sphaerobacter, Arthrobacter, Solidubrobacter, Blastococcus, Aciditerrimonas, Desulfoglaeba*, etc (Figure 6). The abundances of *Burkholderia* were JX (1.54), LQ (1.44), XJ (1.35), HM (1.00), JY (0.92) and LJ (0.15) from high to low, and the abundance of the highest field was 10.27 times that of the lowest field (Table 7).

**Table 8.** The abundances of beneficial microorganism in the six fields at the genus level.

	Genus	HM	JX	JY	LJ	LQ	XJ
CK	*Sphingomonas*	9.16	7.24	5.50	8.69	4.93	4.60
	*Pseudomonas*	1.10	2.87	2.62	1.27	3.19	3.32
	*Rhizorhapis*	0.01	0.01	0.00	0.03	0.01	0.02
Rhizosphere	*Burkholderia*	1.00	1.54	0.92	0.15	1.44	1.35

CK represents the soil samples before cotton planting, and Rhizosphere represent the rhizosphere soil samples of diseased plants.

## 4. Discussion

The interaction between plants and soil microorganisms is of great significance for plant growth and resistance,^13^ and soil microbes were thought to be key drivers of plant-soil feedbacks through affecting plant growth or acting as antagonists of plant pathogens.^21,22^ Microorganisms are an indicator of soil health, especially the inhibition of diseases.^23–25^ High microbial diversity can improve the stability of the community. Higher diversity in soil bacteria is often associated with greater resistance to pathogens.^26,27^ In these six fields, there was no difference in the alpha-diversity index of fungi and bacteria before cotton planting, but there were differences in rhizosphere of Cotton Verticillium wilt diseased plants in some fields, so they showed different degrees of incidence. Previous studies have shown that fungal and bacterial communities in different soils had some difference in alpha-diversity, relative abundance, structure and taxonomic composition, but microbial groups showed similarity in the same habitat, despite different sampling sites; ^3128^ the higher the diversity of soil bacteria, the healthier the cotton plants were,^3229^ but the opposite was true in tomato; ^3330^ the abundance of almost all bacterial OTUs with different abundance in healthy cotton plants was higher than that in diseased cotton, while the abundance of fungal OTUs was the opposite.^7^ In this study, the fungal alpha-diversity index before cotton planting was high in 2 of the 3 fields with severe disease, and the bacterial alpha-diversity index before cotton planting was relatively high in 2 of the 3 fields with mild disease, which was consistent with Wei et al.^7^ Therefore, soil microbial diversity is very important for plant growth.

In the process of plant growth, some soil microorganisms promote plant growth and help plants resist various biological and abiotic stresses, that is, beneficial microorganisms. At present, the recognized beneficial microorganisms included *Bacilli, Gemmatimonadetes, Pseudomonas, Stenotrophomonas*,^31^ *Trichoderma*,^32^ etc. The diversity and community members of *Gammaproteobacterial* have been identified as potential health indicators;^31^ *Sphingomonas* was reported to increase Chinese medicinal plant biomass;^33^ *Actinoplanes* could promote cucumber growth;^34^ *Caulobacter* produced phytohor mones in lavender.^35^ In this study, before cotton planting, the abundances of *Pseudomonas* and *Sphingomonas* in the soil microbial community in the JX field with light disease were significantly higher than that in other fields, which was consistent with that of Köberl et al.^31^ and Ali et al.,^33^ while the abundance of *Rhizorhapis* in the LJ field with heavy disease was significantly higher than that in other fields, which could promote plant nitrogen fixation and may have little correlation with Verticillium wilt resistance. For fungi, before planting, the abundance of *Chaetomium* in XJ field was the highest, and the incidence of Verticillium wilt in XJ field was relatively mild. *Chaetomium globosum* can inhibit a variety of pathogens, inhibit the growth of *V. dahliae*, promote the growth of cotton plants and induce cotton to produce defense response to resist the infection of *V. dahliae*.^36–38^ This study suggested that the application of *Sphingomonas* during cotton planting maybe reduce the occurrence of Verticillium wilt. The next step is to isolate the *Sphingomonas* sp. strains and detect their resistance to Cotton Verticillium wilt.

## 5. Conclusions

Through the analysis of soil microbial communities in six fields with different incidences of Verticillium wilt, there was no difference in fungal and bacterial alpha-diversity indexes before cotton planting, while there were differences in rhizosphere of diseased plants. For fungal beta diversity indexes, there were significant differences in these six fields. The composition of fungi and bacteria in different fields was roughly the same at the genus level, but the abundances of the same genus varied greatly between different fields. *Pseudomonas, Sphingomonas* and *Burkholderia* had higher abundances in the fields with less incidence. In conclusion, we speculate that the lower the fungal diversity index in soil, the higher the bacterial diversity index, and the lighter the incidence of Cotton Verticillium wilt. In addition, *Pseudomonas, Sphingomonas* and *Burkholderia* may improve the resistance of cotton to Verticillium wilt.

## Abbreviations

operational taxonomic units, OTUs; disease index, DI; principal coordinate analyses, PCoA.
